# Artificial intelligence detection of distal radius fractures: a comparison between the convolutional neural network and professional assessments

**DOI:** 10.1080/17453674.2019.1600125

**Published:** 2019-04-03

**Authors:** Kaifeng Gan, Dingli Xu, Yimu Lin, Yandong Shen, Ting Zhang, Keqi Hu, Ke Zhou, Mingguang Bi, Lingxiao Pan, Wei Wu, Yunpeng Liu

**Affiliations:** a Department of Orthopaedics, Ningbo Medical Center, Lihuili Hospital, Ningbo, 315000, China;;; b School of Medicine, Ningbo University, Ningbo, 315000, China;;; c Department of Orthopaedics, Second Affiliated Hospital of Wenzhou Medical University, Wenzhou, 325027, China;;; d Department of Orthopaedics, Second Hospital of Ningbo, Ningbo, 315000, China;;; e Faculty of Electronics & Computer, Zhejiang Wanli University, Ningbo, 315000, China

## Abstract

Background and purpose — Artificial intelligence has rapidly become a powerful method in image analysis with the use of convolutional neural networks (CNNs). We assessed the ability of a CNN, with a fast object detection algorithm previously identifying the regions of interest, to detect distal radius fractures (DRFs) on anterior–posterior (AP) wrist radiographs.

Patients and methods — 2,340 AP wrist radiographs from 2,340 patients were enrolled in this study. We trained the CNN to analyze wrist radiographs in the dataset. Feasibility of the object detection algorithm was evaluated by intersection of the union (IOU). The diagnostic performance of the network was measured by area under the receiver operating characteristics curve (AUC), accuracy, sensitivity, specificity, and Youden Index; the results were compared with those of medical professional groups.

Results — The object detection model achieved a high average IOU, and none of the IOUs had a value less than 0.5. The AUC of the CNN for this test was 0.96. The network had better performance in distinguishing images with DRFs from normal images compared with a group of radiologists in terms of the accuracy, sensitivity, specificity, and Youden Index. The network presented a similar diagnostic performance to that of the orthopedists in terms of these variables.

Interpretation — The network exhibited a diagnostic ability similar to that of the orthopedists and a performance superior to that of the radiologists in distinguishing AP wrist radiographs with DRFs from normal images under limited conditions. Further studies are required to determine the feasibility of applying our method as an auxiliary in clinical practice under extended conditions.

Conventional radiographs remain the primary diagnostic approach to detect distal radius fractures (DRFs) (Mauffrey et al. 2018, Waever et al. 2018). Non-orthopedic surgeons or young radiologists at emergency departments, where urgent decision-making is often required, are usually the first doctors to assess radiographs. Therefore, an accurate and efficient assistant technology in fracture detection is of interest.

Artificial intelligence (AI) is achieving remarkable progress in image interpretation (He et al. 2015, Russakovsky et al. 2015). Since 2012, deep learning, a branch of AI, has rapidly become a powerful method in image analysis with the use of convolutional neural networks (CNNs), which are well suited for analyzing image features (Russakovsky et al. 2015, Lakhani and Sundaram 2017). There are increasing numbers of experimental trials that apply deep learning in medical image analysis in certain fields, including the automated analysis of pulmonary tuberculosis (Lakhani and Sundaram 2017), lung nodules (Hua et al. 2015, Nishio et al. 2018), retinopathy (Ting et al. 2017), gastric cancer (Wang et al. 2018), and dermatological diseases (Li and Shen 2018, Yap et al. 2018, Fujisawa et al. 2019). In the field of traumatic orthopedics, a few studies (Olczak et al. 2017, Chung et al. 2018, Kim and MacKinnon 2018, Urakawa et al. 2019) investigated the experimental applications of deep learning to detect fractures on plain radiographs; all the CNNs adopted showed excellent performance, and some (Chung et al. 2018, Urakawa et al. 2019) had abilities superior to that of humans. To further validate the feasibility of AI as an automatic diagnostic model, we first evaluated the ability of a CNN, with a fast object detection algorithm previously identifying the regions of interest, to detect DRFs on AP wrist radiographs. Second, the diagnostic performances of CNNs were compared with those of radiologists and orthopedists.

## Materials and methods

### Design of study

With the dataset, a fast object detection algorithm based on deep learning was first trained to identify the distal radiuses on AP wrist radiographs as the regions of interest (ROIs). Second, we adopted this fast object detection algorithm, of which the feasibility had been verified by a validation process, to automatically annotate the ROIs on AP wrist radiographs in the training dataset and test dataset. The ROIs were extracted as images, with which a diagnostic CNN model was then trained and tested in detecting the DRFs. The diagnostic performances in terms of accuracies, sensitivities, specificities, and Youden Index were finally compared among the diagnostic CNN model, radiologists, and orthopedists.

### Dataset

2 senior orthopedists with more than 10 years of orthopedic professional experience retrospectively reviewed 2,359 plain wrist radiographs with diagnostic reports from 2,359 adult patients (the inclusion and exclusion criteria for this study are given in the Supplementary data) who underwent radiological examinations at the Medical Center of Ningbo City, Lihuili Hospital, of the Ningbo University School of Medicine, between January 2010 and September 2017 to confirm that each case had an accurate diagnosis (with DRFs or without DRFs). A consensus was achieved in consultation with a third senior orthopedist with 22 years of orthopedic professional experience. For cases in which all 3 orthopedists did not agree, the corresponding wrist CT images were reviewed; CTs were available in most of these cases and a consensus on each case was reached after discussion. 19 controversial cases without CT exams were excluded from the study. 2,340 AP wrist radiographs (1,491 DRF cases and 849 normal wrists) from 2,340 adult patients were ultimately included in the final dataset.

### Data preparation

Each plain AP wrist radiograph, originally stored as a Digital Imaging and Communications in Medicine (DICOM) file, was exported as a Joint Photographic Experts Group (JPEG) file with a matrix size of 600 x 800 pixels from the Picture Archiving and Communication System (PACS) by using eWorld Viewer (TomTaw Tech, Ningbo, China).

For further analyses, 1,491 images with DRFs and 849 images without DRFs (randomized with the Research Randomizer program, http://www. randomizer.org) were randomly divided into an original training dataset of 2,040 images (1,341 images with DRFs and 699 images without DRFs) and a test dataset of 300 images (150 images with DRFs and 150 images without DRFs).

### Training the CNN models

The detailed experimental environment is described in the Appendix (see Supplementary data).

### Training the Faster R-CNN (Region-based CNN)

Faster R-CNN (Ren et al. 2017) technology, one of the fast object detection algorithms based on deep learning, has excellent performance in locating the regions of interest (ROIs) on graphics. In this study, we trained and tested Faster R-CNN as an auxiliary algorithm to the diagnostic CNN model. The detailed training procedure of the Faster R-CNN model is shown in the Appendix.

### Validation of the Faster R-CNN

A regression analysis (Mitra et al. 2018) was used to assess the training process of Faster R-CNN. The mean square error (MSE) (Kumar et al. 2018) was calculated to measure the loss of Faster R-CNN in the automatic annotation of the ROI.

**Figure 1.**
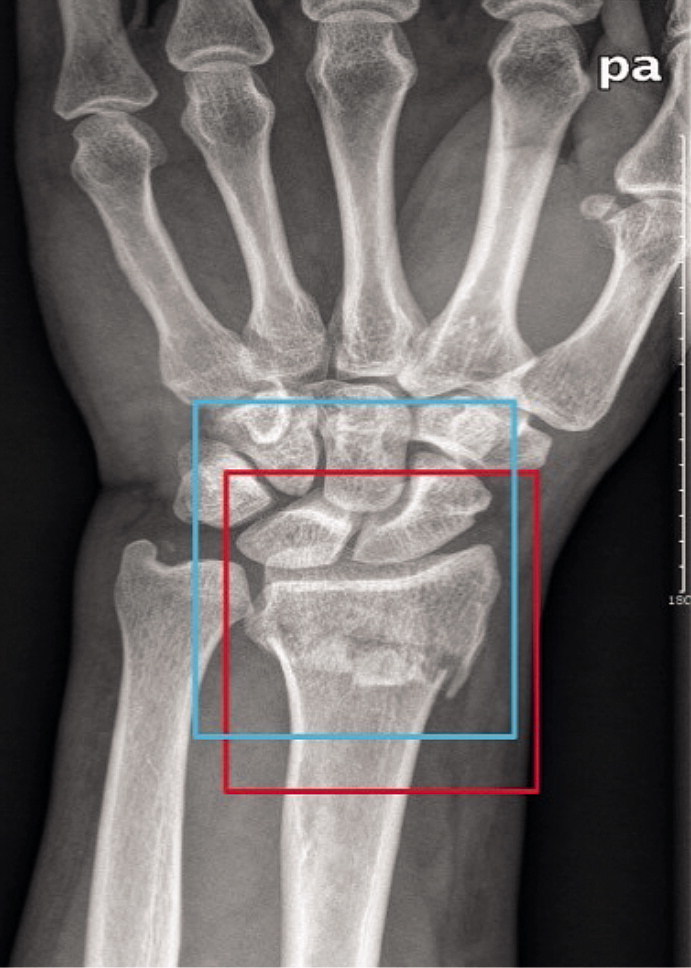
A wrist radiograph was manually annotated with a red rectangle as the ground truth bound and automatically annotated with a blue rectangle as the candidate bound. The red rectangle and blue rectangle represent edges of the region of interest (ROI) detected by the orthopedists and edges of the ROI detected by Faster R-CNN, respectively.

**Figure 2.**
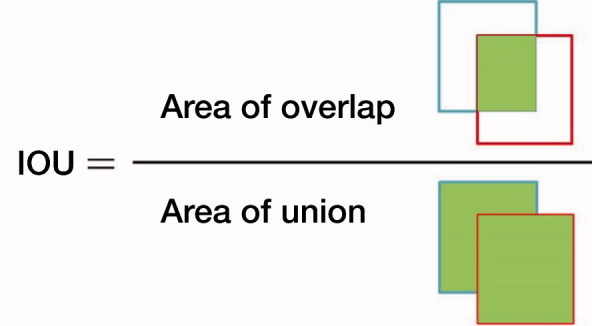
The formula with which the Intersection of the Union (IOU) was calculated.

The test dataset of 300 images, including 150 images with fractures and 150 images without fractures, was used to evaluate the capacity of the trained Faster R-CNN model in automatic annotation of the ROI on images. First, using LabelImg (https://github.com/tzutalin/labelImg), 2 orthopedists with more than 5 years of orthopedic professional experience annotated each image’s ROI as the ground truth bounds (GTBs), in which the whole distal radius was definitely encased. Then, a candidate boundary (CB) on each image with a GTB was annotated as the automatically detected ROI by the trained Faster R-CNN (Figure 1). The matrix sizes of the identified ROIs ranged from 207 to 223 pixels in width and from 208 to 231 pixels in height, respectively. The Intersection of the Union (IOU) (Mitra et al. 2018) was calculated, as illustrated in Figure 2, to statistically evaluate the trained Faster R-CNN, with a value greater than 0.5 indicating success in detecting the ROI on an image.

### Training the diagnostic CNN model

We used Inception-v4 (Szegedy et al. 2017) as the diagnostic model, which has achieved state-of-the-art results in recent image classification contests.

In this study, only the images’ ROIs automatically annotated by Faster R-CNN were used as the recognition targets; after the ROI was extracted, the rest region on each initial image was discarded as unnecessary interference factors and noises. Since the areas where a DRF would occur were focused on, the Inception-v4 model’s training process of distinguishing images with fractures from normal images was much faster and more accurate than analyzing the entire image.

First, each initial image in the original training dataset, including 1,341 images with DRFs and 699 images without DRFs, was automatically annotated by the trained Faster R-CNN. The result of the annotations on 2,040 images was reviewed by 2 orthopedists, and each distal radius region was then confirmed to be appropriately encased in the bounds.

**Figure 3.**
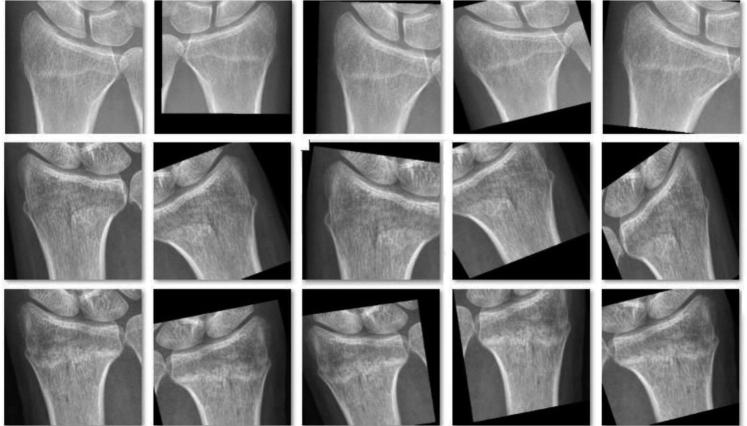
A typical example of the augmentation on 1 image from the annotated training dataset during the training course of Inception-v4.

**Figure 4.**
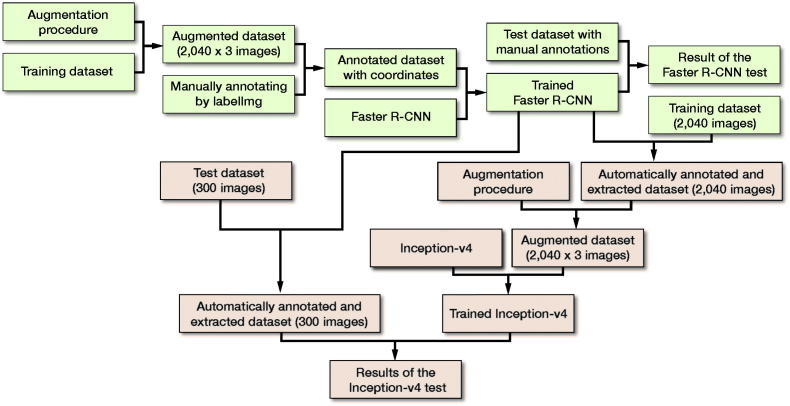
Flow diagram of the training and test courses of Faster R-CNN (shown in a green) and Inception-v4 (shown in a red).

The ROIs extracted from all the annotated images were resized to 200 x 200 pixels, and stored as JPEG files, which were then augmented via the same technique as that used in the training of Faster R-CNN (Figure 3). Finally, there were 6,120 images in the data pool as the final training dataset for the Inception-v4 model, including 4,023 images with DRFs and 2,097 images without DRFs; 15% of the dataset was randomly selected into the validation dataset. The summary of the training course is illustrated in Figure 4. The detailed training procedure of the Inception-v4 model is shown in the Appendix.

### Evaluation of the diagnostic performance of Inception-v4

First, each initial image in the original test dataset, including 150 images with DRFs and 150 images without DRFs, was automatically annotated by the trained Faster R-CNN. The result of the annotations on 300 images was reviewed by 2 orthopedists, and each distal radius region was then confirmed to be appropriately encased in the bounds. The ROIs extracted from the 300 annotated images were all resized to 200 x 200 pixels, and stored as JPEG files, consisting of a new test dataset.

The final analysis of the trained Inception-v4 model was performed using the new test dataset of 300 images to inspect its ability to discern images with fractures from the normal images. Each image was analyzed using the trained Inception-v4 model, which resulted in a score representing the likelihood that the image would be classified as “with a DRF” or “without a DRF.” This score had a continuous value between 0 and 1. The receiver operating characteristic (ROC) curve was generated using a Python script (https://www.python.org), and the AUC was determined.

### Evaluation of the performance of the medical professionals

We set up a group of radiologists and a group of orthopedists to compare their results with those of the CNN to evaluate its diagnostic performance. The groups consisted of 3 radiologists who had at least 3 years of radiological professional experience and had passed the intermediate certificate exams and 3 orthopedists (none of whom participated in the validating process of review) with more than 5 years of orthopedic professional experience. The detailed procedure is described in the Appendix. Each image in the new test dataset was diagnosed as either “with a DRF” or “without a DRF.” In situations where disagreements arose in the same group regarding the diagnoses, the final decisions were made by a majority vote.

### Comparison of the results of Inception-v4 and those of the medical professionals

After the ROC curve of Inception-v4 had been generated, the diagnostic cut-off at a threshold designed to maximize the Youden Index was set, and sensitivity, specificity, and accuracy of the Inception-v4 model were then calculated and statistically compared with these of the human groups.

### Statistics

The SPSS software (version 22.0, IBM Corp, Armonk, NY, USA) was used to perform all statistical analyses. The demographic characteristics of all patients enrolled in this study are presented as mean (95% confidence intervals (CIs)) for age and count (percentage) for sex. P-values were derived from 1-way analysis of variance for age and chi-square tests for sex. The significance level was set at p < 0.05.

The IOU of Faster R-CNN and AUC of Inception-v4 were calculated and described in terms of the means and CIs. CIs of the distributions for the 4 kinds of outcomes and for the differences in the outcomes between the CNN model and each human group were computed via bootstrapping with 10,000 bootstraps. Comparisons between the CNN model and each human group were performed using a 1-way analysis of variance, followed by Dunnett’s test for multiple comparison with the significance level set at p < 0.05.

### Ethics, funding, and potential conflicts of interest

The Ningbo Lihuili Hospital Ethics Committee approved the study (LH2018-039). Financial support for the study was from the Ningbo Natural Science Fund (No.2018A610164). All authors declare no conflicts of interest.

## Results

### Demographic data of the included patients

All of the patient radiographs (1,366 men and 974 women) were kept anonymous throughout this study. The patients’ mean age at the time they took the radiographs was 48 years (20–88). No statistically significant difference was found in age (p = 0.4) between the group of patients with fractures and group of patients without fractures, but there was significant difference in sex between the 2 groups (p < 0.01) (Table 1).

**Table 1.** Demographic data of the whole dataset with 2,340 patients enrolled in this study

Factor	Patients with DRFs (n = 1,491)	Patients without DRFs (n = 849)	Total (n = 2,340)	Comparison (p-value)
Age, mean (CI)	48 (48–49)	48 (47–49)	48 (48–49)	0.4
Sex, n (%)				
Male	833 (56)	533 (63)	1,366 (58)	< 0.01
Female	658 (44)	316 (37)	974 (42)	

DRFs = distal radius fractures.

CI = 95% confidence interval.

P-values were derived from 1-way analysis of variance for age and chi-square tests for sex.

### Performance of Faster R-CNN

The learning courses of Faster R-CNN in the final training and validation datasets are shown in the Appendix. In the test dataset, the average IOU value of Faster R-CNN was 0.87 (CI 0.86–0.87), and none of the IOU values was less than 0.5. 2 orthopedists reviewed each annotated CB, which was confirmed by encasing the whole distal radius on each image.

### Performance of the Inception-v4 model

The learning courses of Inception-v4 in the final training and validation datasets are shown in the Appendix.

**Figure 5.**
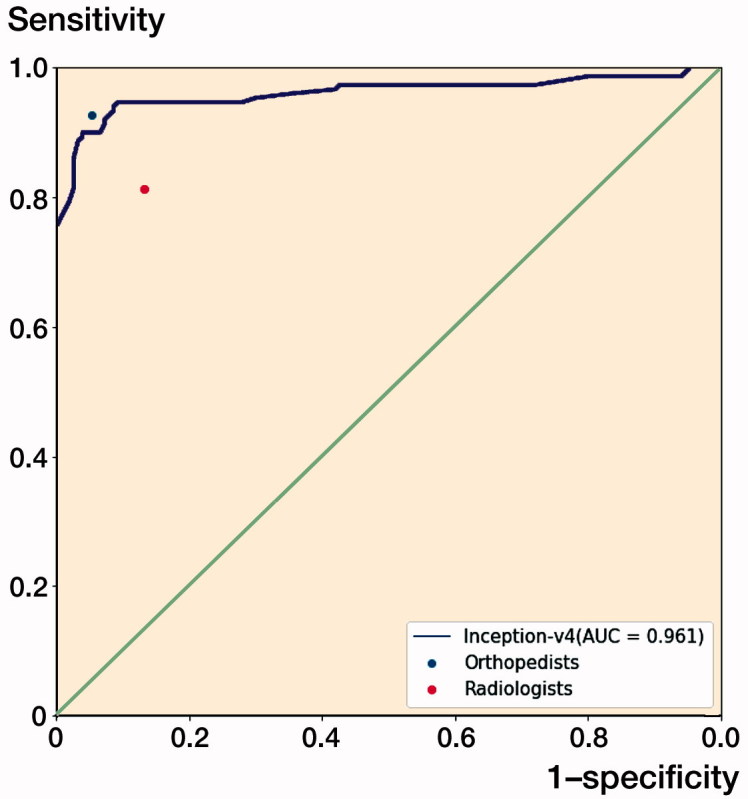
The receiver operating characteristic (ROC) curve for the test output of the Inception-v4 model. The dots on the plot represent the sensitivity and 1-specificity of the human groups (the blue dot represents the orthopedists’ group; the red dot represents the radiologists’ group). The sensitivity/1-specificity dot of the radiologists’ group lies below the ROC curve of the Inception-v4 model, and the sensitivity/1-specificity dot of the orthopedists’ group lies above the ROC curve of the Inception-v4 model.

The ROC curve for the test output of Inception-v4 is plotted in Figure 5, and the AUC was 0.96 (CI 0.94–0.99). At the optimal cut-off point, the value of the threshold was 0.64.

### Comparison between the Inception-v4 model and human performance

The model showed a superior capacity compared with the radiologists’ group to distinguish images with DRFs from normal images in terms of accuracy, sensitivity, specificity, and Youden Index. The CNN model presented a similar diagnostic capability to that of the orthopedists in terms of the outcomes (Tables 2 and 3).

**Table 3.** Performance differences in the outcomes between Inception-v4 and each human group

Factor	Differences between orthopedists Difference^a^ (CI)	Inception-v4 and radiologists Difference^b^ (CI)
Accuracy (%)	–1 (–5 to 3)	9 (3–15)
Sensitivity (%)	–3 (–9 to 4)	9 (1–16)
Specificity (%)	1 (–5 to 7)	9 (3–16)
Youden Index	–0.01 (–0.09 to 0.06)	0.18 (8–27)

CI = 95% confidence interval.

a Difference = (mean of the outcome of Inception-v4) – (mean of the outcome of orthopedists).

b Difference = (mean of the outcome of Inception-v4) – (mean of the outcome of radiologists).

**Table 2.** Diagnostic performance of the model and human groups

Factor	Inception-v4	Orthopedists	Radiologists	F-value	p-value
Accuracy (%) [CI]	279/300 (93) [90–96]	281/300 (94) [91–96]	252/300 (84) [80–88] a	10.19	< 0.001
Sensitivity (%) [CI]	135/150 (90) [85–95]	139/150 (93) [89–97]	122/150 (81) [75–87]^a^	5.07	0.007
Specificity (%) [CI]	144/150 (96) [93–99]	142/150 (95) [91–98]	130/150 (87) [81–92]^a^	4.82	0.009
Youden Index (CI)	0.86 (0.80–0.91)	0.87 (0.82–0.93)	0.68 (0.61–0.75)^a^	11.62	< 0.001

CI = 95% confidence interval.

a Statistically significant in a comparison of Inception-v4 and each human group (results from Dunnett’s test).

## Discussion

In our study, both deep learning models demonstrated an excellent ability to recognize image traits in wrist radiographs. The trained Faster R-CNN, which had a 100% success rate in automatically annotating the ROIs on images from the test dataset, acted as a valid auxiliary algorithm to the Inception-v4 model, which was trained to distinguish images with DRFs from normal images. The Inception-v4 model exhibited a similar diagnostic capability to that of the orthopedists and superior performance to that of the radiologists.

Previous studies investigating the feasibility of applying CNNs to detect fractures on radiographs showed promising results, consistent with those of our study. Kim and MacKinnon (2018) trained Inception-v3 to recognize wrist fractures on lateral wrist radiographs; their results showed that the value of AUC was 0.954 and the maximized values of the sensitivity and specificity were 0.9 and 0.88, respectively. Olczak et al. (2017) performed a study in which a Visual Geometry Group 16-layer (VGG_16) network was trained to detect fractures on hand, wrist, and ankle radiographs with an accuracy of 83%, similar to the performance of the radiologists (who had an accuracy of 82%). Chung et al. (2018) evaluated the ability of the Residual Network (ResNet) model to detect and classify proximal humerus fractures using shoulder radiographs. The CNN showed superior top-1 accuracy, an accuracy of 96%, which was greater than that of the orthopedists (93%). Urakawa et al. (2019) conducted a study in which they compared the capacities of the VGG_16 network and orthopedic surgeons in detecting intertrochanteric fractures on radiographs, revealing the diagnostic performance of the CNN; the CNN had an accuracy of 96%, which exceeded that of orthopedic surgeons, who had an accuracy of 92%. All the previous studies mentioned prepared the images that were used in the training datasets and test datasets by manually cropping them into certain matrix sizes before the images were input into the CNNs. Since the images were uniform and had concentrated matrix sizes, the ROIs on the images were recognized faster and more accurately by the deep learning models, thereby remarkably improving the efficiency of the CNNs in the learning and test procedures. We employed and trained the Faster R-CNN model to automatically annotate the ROIs on images as a reliable substitution for manual cropping, which resulted in a low processing time and decreased bias (Urakawa et al. 2019). There is a great potential for the Inception-v4 model to be combined with Faster R-CNN to detect DRFs in clinical practice, where wrist radiographs with both ROIs and irrelevant regions are presented.

**Figure 6.**
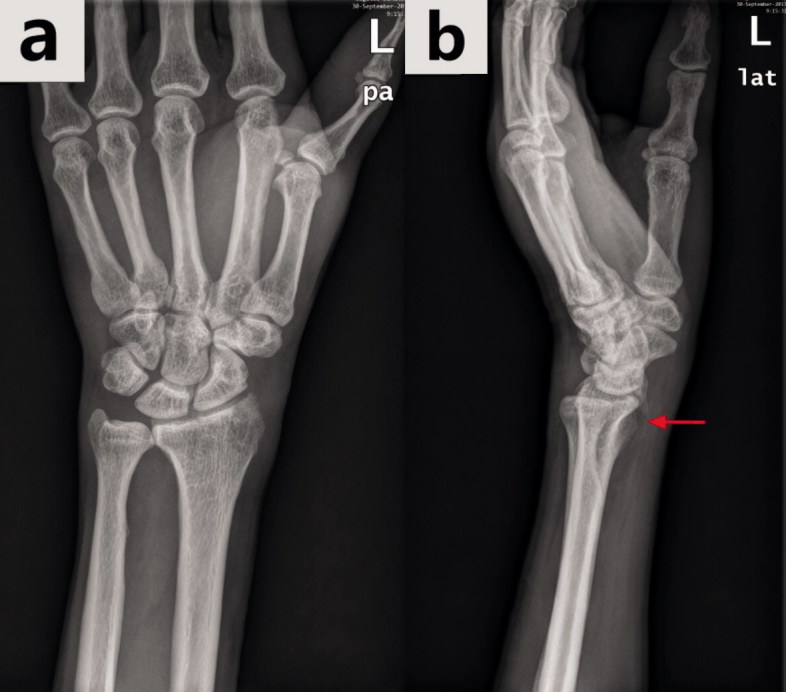
The same wrist with a DRF in the anterior–posterior view radiograph (a) and in the lateral view radiograph (b). The hidden DRF in the anterior–posterior view was apparent in the lateral view (the fracture is shown by the red arrow).

We plotted the ROC curve for the test output; at the optimal cut-off point, Inception-v4 showed a sensitivity of 90% (135/150), which is much lower than the specificity (96%). In such conditions, some wrist AP radiographs with DRFs appeared to be misdiagnosed as normal images by the model, resulting in a delay in essential treatment for injured patients. After reviewing the 15 images with fractures, whose predicted values by the Inception-v4 model were less than the threshold (0.64), we found that 5 of them displayed an absence of apparent fracture traits (e.g., fracture lines or fracture fragment displacement). However, such traits were visible on the lateral radiographs corresponding to the AP images, as shown in Figure 6. The ensemble of model analyses using AP and lateral radiographs has the potential to enhance the sensitivity in fracture detection.

In the total enrolled dataset, there was a statistically significant difference between the group of patients with DRFs and the group of patients without DRFs in sex (Table 1). This difference may affect the results of training and testing the CNN models due to difference in anatomical traits in distal radius between the male and the female (Oppermann et al. 2015). But we cannot declare to what extent the effect of gender difference would be on the results in this study.

There are several limitations in our study. First, the original sample size in our dataset was small. However, we did not increase the original sample size by obtaining new wrist radiographs from other medical centers to maintain uniformity in the image quality. This small sample size might restrict the improvement of the CNN’s performance in the training and test procedures. Data augmentation was used to address the sample size issue, since it can reduce over-fitting and improve performance (Wong et al. 2016). Second, the assessment of the diagnostic performance of the deep learning models was based on anterior–posterior wrist radiographs, so the procedure may not represent a practical scenario. Typically, at least 2 wrist radiographs (an anterior–posterior image and a lateral image) are obtained by the reader to review. We will investigate whether the performance of the CNN would improve when the dataset consists of anterior–posterior wrist radiographs and matched lateral radiographs in our next planned project. Finally, we trained Inception-v4 to simply distinguish images with DRFs from normal images. The deep learning algorithm could accurately classify proximal humerus fractures based on Neer’s classification on shoulder radiographs (Chung et al. 2018), so as part of our next project we will train the CNN model to classify DRFs based on 1 particular fracture classification system.

In summary, the network exhibited a similar diagnostic capability to that of the orthopedists and a superior performance to that of the radiologists in distinguishing AP wrist radiographs with DRFs from normal radiographs under limited conditions. Further studies are required to determine the feasibility of applying the diagnostic network with the object detection algorithm as an auxiliary in clinical practice under extended conditions.

## Supplementary Material

Supplemental Material

## Figures and Tables

**Figure 7.**
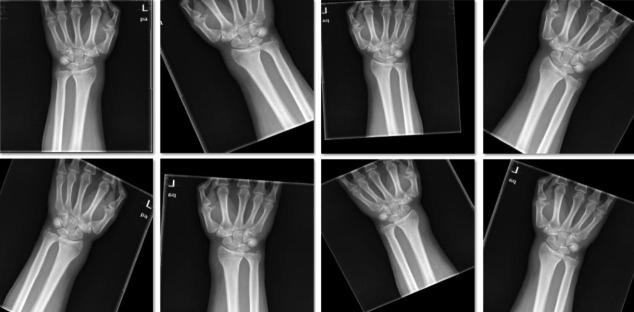
A typical example of the augmentation on 1 image from the training dataset during the training of Faster R-CNN (the top left image is the original one).

**Figure 8.**
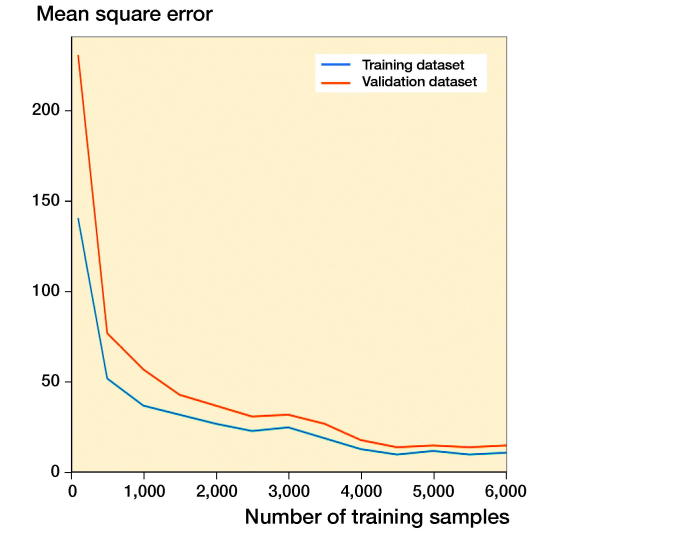
The training processes of Faster R-CNN with respect to the training sample in the training dataset and validation dataset. The mean square error (MSE) with value close to 0 indicates the accurate performance of the model.

**Figure 9.**
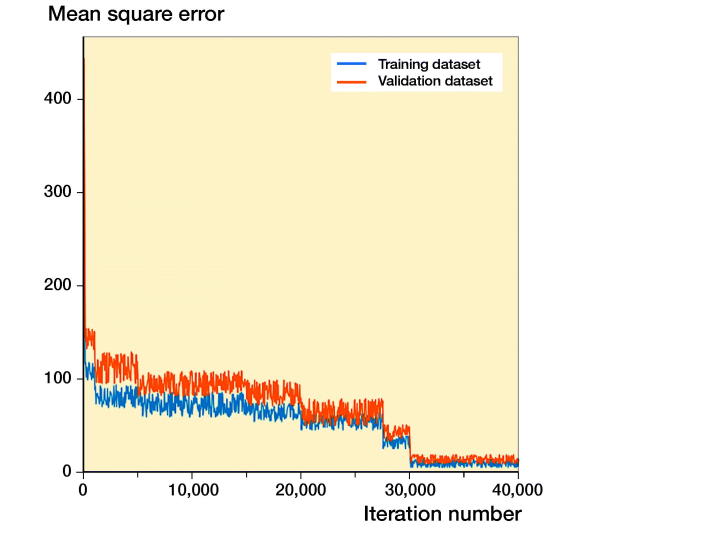
The training processes of Faster R-CNN with respect to the iteration number in the training dataset and validation dataset. The mean square error (MSE) with value close to 0 indicates the accurate performance of the model.

**Figure 10.**
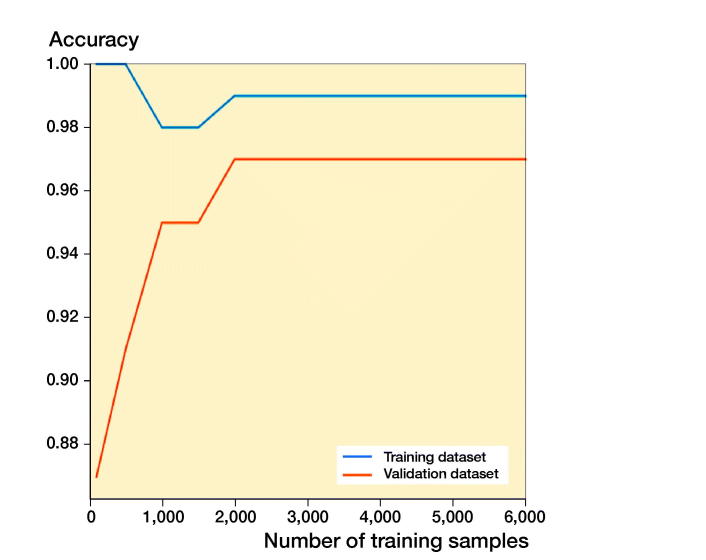
The training processes of the Inception-v4 model with respect to the training sample in the training dataset and validation dataset.

**Figure 11.**
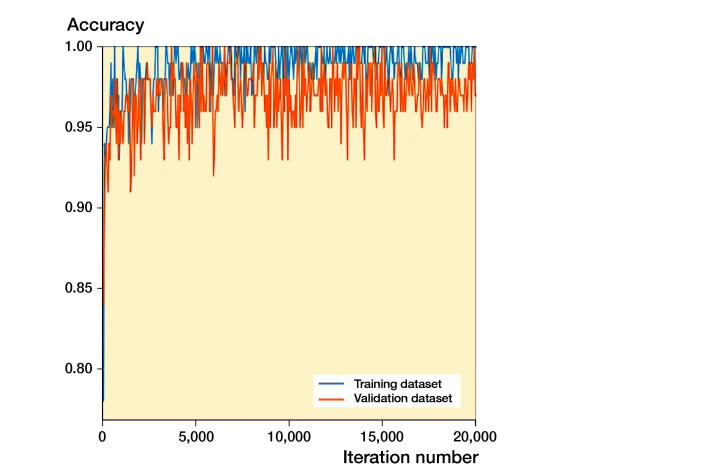
The training processes of the Inception-v4 model with respect to the iteration number in the training dataset and validation dataset.

## Supplementary data

The inclusion and exclusion criteria for this study and the Appendix are available as Supplementary data in the online version of this article, http://dx.doi.org/10.1080/17453674.2019.1600125

Conception and design of this study: KG, YL, and DX. Collection and preparation of the datasets: KG, DX, YL, YS, TZ, KH, and KZ. Training and testing of models: YL, DX, MB, YS, TZ, and KH. Analysis and interpretation of the results and data: KG, YL, LP, and WW.

*Acta* thanks Martin Gerdin Wärnberg, Max Gordon and Takaaki Urakawa for help with peer review of this study.

## APPENDIX

### Materials and methods

#### Dataset

Inclusion criteria for this study were (1) it was his/her first visit in our hospital for radiological examination; (2) at least both standard anterior–posterior and lateral wrist radiographs had been taken at this visit, and the report was available. Exclusion criteria were: (1) casts or splints were present in the wrist radiographs; (2) distal ulna fractures, fractures of carpal bones, or any dislocations in wrist were present in the radiographs.

#### Training the CNN models

##### Experimental environment

We ran the Google open source AI platform TensorFlow 1.11.0 (https://www.tensorflow.org) on the Ubuntu 16.04 operation system (http://www.ubuntu.com) with an NVIDIA Titan X GPU (CUDA 9.0 and cuDNN 7.0) (http://developer.nvidia.com), 12 GB VRAM, 8 GB RAM, and Intel Core i7 CPU@2.5GHz (https://www.intel.com/).

#### Training the Faster R-CNN (Region-based CNN)

The original training dataset, which included 1,341 images with DRFs and 699 images without DRFs, was used in training Faster R-CNN to detect the distal radius regions as the ROIs on the images in this study. The initial images in the original training dataset were augmented by a random horizontal inversion, random offset within 10% of the height and width, random rotation within 30 degrees, 10% random scaling, and 15% random shearing (Figure 7). In total, there were 6,120 images in the data pool that comprised the final training dataset, including 4,023 images with DRFs and 2,097 images without DRFs; 15% of the dataset was randomly selected into the validation dataset. 2 orthopedists with more than 5 years of orthopedic professional experience applied LabelImg (https://github.com/tzutalin/labelImg), which was used as an object detection tagging tool, to manually annotate the ROI on each image from the final training dataset (Figure 1). The ROI coordinates, which were generated automatically as soon as each annotation was made via LabelImg, were recorded at the same time. While training Faster R-CNN, we input the original images and the matched coordinates of the ROIs. The summary of the training course is illustrated in Figure 4.

The training procedure of the Faster R-CNN model was featured with the parameters as follows. Optimizer, stochastic gradient descent; batch size, 100; dropout, 0.5; 40,000 iterations; initial learning rate, 0.001; Learning Rate = Learning Rate * 1/(1 + decay * epoch); weight decay, 0.0005. The best network parameters were adopted in the test process with the validation datasets.

#### Training the diagnostic CNN model

Training procedure of the Inception-v4 model was featured with the parameters as follows. Optimizer, stochastic gradient descent; batch size, 100; dropout, 0.5; 20,000 iterations; initial learning rate, 0.001; learning rate decay type, fixed. The best network parameters were adopted in the test process with the validation datasets.

#### Evaluation of the performance of the medical professionals

Each group performed its final analysis separately on the same liquid crystal display monitor (Nio Color 2MP LED, BARCO, Belgium) (Resolution, 1600 x 1200; Brightness, 400 cd/m^2^; contrast ratio, 1,400:1). Readers in each group reviewed the resized 300 images from the new test dataset at the same resolution as the CNN. Adjustments in the zooming, brightness, or contrast of the displayed images were performed by the readers when the fracture features were indistinct in default mode.

### Results

#### Performance of Faster R-CNN

The learning courses of Faster R-CNN in the final training and validation datasets are shown in Figures 8 and 9. The learning curve (Figure 8) shows the relation between sample sizes and accuracies of training and validation processes, and the training curve (Figure 9) shows the relation between number of iterations and accuracies of training and validation processes.

#### Performance of the Inception-v4 model

The learning courses of Inception-v4 in the final training and validation datasets are shown in Figures 10 and 11. The learning curve (Figure 10) shows the relation between sample sizes and accuracies of training and validation processes, and the training curve (Figure 11) shows the relation between number of iterations and accuracies of training and validation processes.
