# Supplementary material for: Artificial intelligence detection of distal radius fractures: a comparison between the convolutional neural network and professional assessments
Source: Acta Orthop. 2019 Apr 3;90(4):394–400. doi: 10.1080/17453674.2019.1600125 (PMC6718190; doi:10.1080/17453674.2019.1600125)
Supplement: Supplemental Material [file IORT_A_1600125_SM2752.pdf]

Supplementary data

APPENDIX

Materials and methods

Dataset

Inclusion criteria for this study were (1) it was his/her first visit in our hospital for radiological examination; (2) at least both standard anterior–posterior and lateral wrist radiographs had been taken at this visit, and the report was available. Exclusion criteria were: (1) casts or splints were present in the wrist radiographs; (2) distal ulna fractures, fractures of carpal bones, or any dislocations in wrist were present in the radiographs.

Training the CNN models

Experimental environment

We ran the Google open source AI platform TensorFlow 1.11.0 (https://www.tensorflow.org) on the Ubuntu 16.04 operation system (http://www.ubuntu.com) with an NVIDIA Titan X GPU (CUDA 9.0 and cuDNN 7.0) (http://developer.nvidia.com), 12 GB VRAM, 8 GB RAM, and Intel Core i7 CPU@2.5GHz (https://www.intel.com/).

Training the Faster R-CNN (Region-based CNN)

The original training dataset, which included 1,341 images with DRFs and 699 images without DRFs, was used in training Faster R-CNN to detect the distal radius regions as the ROIs on the images in this study. The initial images in the original training dataset were augmented by a random horizontal inversion, random offset within 10% of the height and width, random rotation within 30 degrees, 10% random scaling, and 15% random shearing (Figure 7). In total, there were 6,120 images in the data pool that comprised the final training dataset, including 4,023 images with DRFs and 2,097 images without DRFs; 15% of the dataset was randomly selected into the validation dataset. 2 orthopedists with more than 5 years of orthopedic professional experience applied LabelImg (https://github.com/tzutalin/labelImg), which was used as an object detection tagging tool, to manually annotate the ROI on each image from the final training dataset (Figure 1). The ROI coordinates, which were generated automatically as soon as each annotation was made via LabelImg, were recorded at the same time. While training Faster R-CNN, we input the original images and the matched coordinates of the ROIs. The summary of the training course is illustrated in Figure 4.

The training procedure of the Faster R-CNN model was featured with the parameters as follows. Optimizer, stochastic gradient descent; batch size, 100; dropout, 0.5; 40,000 iterations; initial learning rate, 0.001; Learning Rate = Learning Rate * 1/(1 + decay * epoch); weight decay, 0.0005. The best network parameters were adopted in the test process with the validation datasets.

Training the diagnostic CNN model

Training procedure of the Inception-v4 model was featured with the parameters as follows. Optimizer, stochastic gradient descent; batch size, 100; dropout, 0.5; 20,000 iterations; initial learning rate, 0.001; learning rate decay type, fixed. The best network parameters were adopted in the test process with the validation datasets.

Evaluation of the performance of the medical professionals

Each group performed its final analysis separately on the same liquid crystal display monitor (Nio Color 2MP LED, BARCO, Belgium) (Resolution, 1600 x 1200; Brightness, 400 cd/m^2; contrast ratio, 1,400:1). Readers in each group reviewed the resized 300 images from the new test dataset at the same resolution as the CNN. Adjustments in the zooming, brightness, or contrast of the displayed images were performed by the readers when the fracture features were indistinct in default mode.

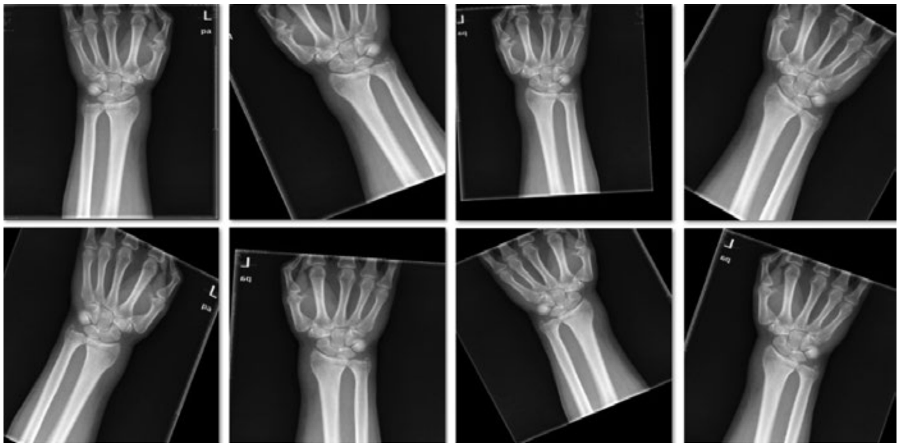

Figure 7. A typical example of the augmentation on 1 image from the training dataset during the training of Faster R-CNN (the top left image is the original one).

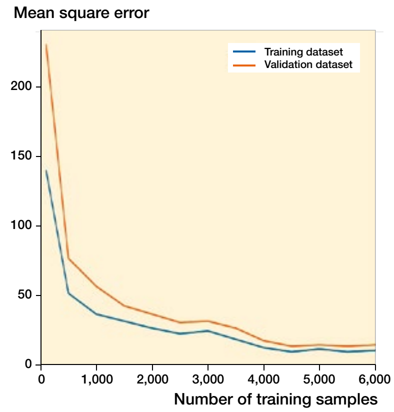

Figure 8. The training processes of Faster R-CNN with respect to the training sample in the training dataset and validation dataset. The mean square error (MSE) with value close to 0 indicates the accurate performance of the model.

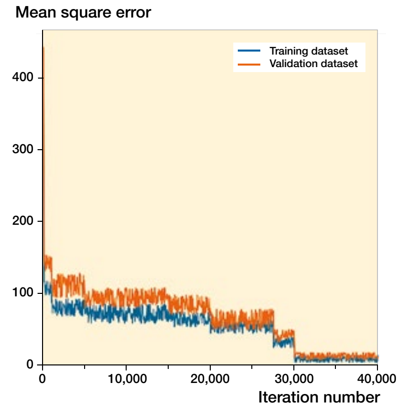

Figure 9. The training processes of Faster R-CNN with respect to the iteration number in the training dataset and validation dataset. The mean square error (MSE) with value close to 0 indicates the accurate performance of the model.

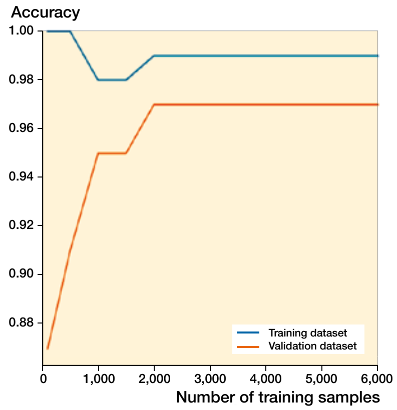

Figure 10. The training processes of the Inception-v4 model with respect to the training sample in the training dataset and validation dataset.

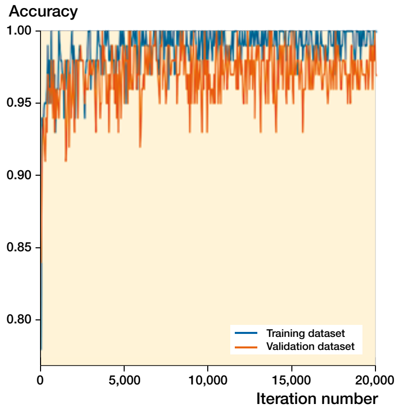

Figure 11. The training processes of the Inception-v4 model with respect to the iteration number in the training dataset and validation dataset.

Results

Performance of Faster R-CNN

The learning courses of Faster R-CNN in the final training and validation datasets are shown in Figures 8 and 9. The learning curve (Figure 8) shows the relation between sample sizes and accuracies of training and validation processes, and the training curve (Figure 9) shows the relation between number of iterations and accuracies of training and validation processes.

Performance of the Inception-v4 model

The learning courses of Inception-v4 in the final training and validation datasets are shown in Figures 10 and 11. The learning curve (Figure 10) shows the relation between sample sizes and accuracies of training and validation processes, and the training curve (Figure 11) shows the relation between number of iterations and accuracies of training and validation processes.
